# Reduced ‘fates of the body’ and ‘production of value for others’ in the global garment industry: Thinking with Berlant on eating and hunger during the COVID‐19 pandemic

**DOI:** 10.1111/geoj.12454

**Published:** 2022-06-19

**Authors:** Katherine Brickell, Sabina Lawreniuk

**Affiliations:** ^1^ Department of Geography, Royal Holloway University of London Egham UK; ^2^ School of Geography University of Nottingham Nottingham UK

**Keywords:** Cambodia, garment, hunger, labour, pandemic

## Abstract

During the COVID‐19 pandemic, suspended and laid off garment workers struggled on severely reduced incomes to meet the cost of food for themselves and their families. It is in this context of ‘double crisis’ that our commentary focuses on the diminished eating and reduced bodily fates of garment workers during the COVID‐19 pandemic. We argue that thinking with Berlant encourages and supports scholars to contemplate and articulate how the ‘production of value for others’ can reduce the ‘fates of the body’ of those living and labouring at the sharp end of the capitalist system.

## 1 INTRODUCTION

As the COVID‐19 pandemic took hold in early 2020, #PAYYOURWORKERS became widely circulated by trade unions and labour rights organisations campaigning for immediate support for garment workers globally. Photographed in the modest rental rooms which they both sleep and cook in, affected Cambodian garment workers held up the rallying hashtag on hand‐written cardboard signs. The campaign's landing page (https://www.payyourworkers.org) featured one such worker and led with the message: ‘Garment workers can't feed their families. Fashion brands are turning their backs’. Well known brands had contravened contractual obligations to cancel orders and withhold payments as demand for clothing in the US and Europe plummeted and raw material supply chains were severed. Between March 2020 and March 2021, it is estimated that garment workers were deprived of $11.85 billion in income (Clean Clothes Campaign, 2021). During this time, millions of workers worldwide were suspended from their positions with little or no pay and others permanently lost their jobs as factories closed (Lawreniuk, 2020). Flexibilisation, already synonymous with the global garment industry, was being intensified at speed by brand responses to the pandemic, which prioritised the profit bottom line over workers' welfare.

Two‐years of longitudinal research across the pandemic with the same cohort of 200 garment workers in Cambodia sought to understand and amplify their experiences of navigating its economic impacts (see www.refashionstudy.org). Since March 2020, around 150,000 of the Cambodian garment industry workforce were suspended or laid off, as the effects of consumer and manufacturing lockdowns spread through global supply chains (Arnold, 2021). A ‘double crisis’ emerged, garment workers losing their employment and income, and hunger amassing amongst a population compelled to adjust and reduce their consumption of food to make ends meet (Mishra & Rampal, 2020, p. 1). Coming into the COVID‐19 pandemic, Cambodia already had levels of hunger that were designated ‘serious’, ranking 76th out of 107 countries in the 2020 Global Hunger Index. Studies of the garment sector in Cambodia had also found that workers were medically malnourished, consuming an average intake of 1598 calories per day, which is half the recommended amount for a woman working in an industrial context (Labour Behind the Label, 2013).

That garment workers are going hungry with the COVID‐19 pandemic in Cambodia (Brickell et al., 2022) is an experience more widely shared in other countries, including Bangladesh, El Salvador, Ethiopia, Haiti, India, Indonesia, Lesotho and Myanmar (Kyritsis et al., 2020). In Cambodia, the country's two other most important economic sectors, construction and tourism, were also heavily impacted and in 2021 strict lockdowns left what was described by some commentators in the Cambodian press as a humanitarian crisis with whole areas of Phnom Penh barricaded in for weeks on end. It was garment workers and their translocal households, however, for whom the issue of food deprivation and hunger has been most systematically encountered in the country. This is because the garment sector in Cambodia sits at the epicentre of the national economy, employing at its pre‐pandemic peak around 1 million workers (Lawreniuk & Parsons, 2020). Moreover, garment factory positions in urban areas had become vital stalwarts in the livelihood profiles not only of women workers themselves, but also their rural‐based households, whose members rely upon their wage‐financed remittances to pay for everyday expenditures, including food.

## 2 REDUCED FATES OF THE BODY DURING THE COVID‐19 PANDEMIC

During the COVID‐19 pandemic, garment workers therefore struggled to meet the cost of food for themselves and their families on severely reduced incomes. It is in this context of ‘double crisis’ that our commentary focuses on the diminished eating and reduced bodily fates of garment workers during the COVID‐19 pandemic. To aid this process, we draw upon Berlant's (2007, p. 768) thinking on eating and obesity as linked to the logics of capitalism and the ‘reduced fate of the body under regimes of production of value for others’. Taking the United States as the analytical pivot, Berlant brings into tension the life‐building dimensions of food consumption against the life‐damaging role of the global processed‐food regime in the country's obesity epidemic. The regime's hugely profitable circulation of unhealthy commodities, in concert with its political and marketing strategies to undermine public health contributes, Berlant argues, to the disproportionate ‘pressure of obesity’ on the organs and skeletons of poor communities and communities of colour. For Berlant then, the obesity epidemic, is ‘a way of talking about the destruction of life, or bodies, imaginaries, and environments by and under contemporary regimes of capital’ (Berlant, 2007, p. 764).^1^ Thinking with Berlant encourages and supports scholars to contemplate and articulate how the ‘production of value for others’ can reduce the ‘fates of the body’ of those living and labouring at the sharp end of the capitalist system.

The ReFashion research underscores the salience of Berlant's (2007, pp. 766–7) contention that ‘mass emaciation and obesity are mirror symptoms of the malnourishment of the poor through the contemporary world’. Despite our concord here, they under‐explore the wearing out and displeasure that comes from having to restrict food consumption. Experiences and feelings of ‘wearing out’ and depletion are replete in hunger and linked anxieties tied to survival in times of economic crisis especially. As one worker's experience speaks to in the ReFashion study:

I begged them not to suspend me. I begged them for work because I had no money … I was screaming and crying loudly … I'm having to endure eating salt and fermented fish only until I have a full salary. (Chantou, suspended Cambodian garment worker)

Even for garment workers who were not suspended or permanently dismissed, their encounters of harsher working conditions, higher production targets, more irregular or reduced pay, and inflation all contributed to a chronic situation of hunger for workers and their families during the COVID‐19 pandemic. Eating might indeed be a form of ‘ballast against wearing out’ (Berlant, 2007, pp. 778–779), but it is not guaranteed.

Over 15 months from January 2020, the average monthly wage fell from a peak of $237 per month in January 2020 to lows of $145 and $147 per month in May and October 2020, respectively. Before the pandemic, on a typical day, the mean cost of food that workers' household eats was US$4.07 but this had dropped to US$3.67 when our quantitative survey was administered in November–December 2020. At this time, 23% of women reported eating less meat than before COVID‐19, 13% reported eating fewer vegetables, and 11% reported eating less fish. For workers who caught COVID‐19 and were mandated to mass quarantine centres, several report putting on much needed weight given the provision of food there:

There is more than enough food. They provide it at a regular time, two options and with dessert too. We rarely eat this kind of food. As you know, workers can only eat 3000 riels (US$0.75) of food per meal, and which has little protein and is plain tasting. While I received treatment for the COVID‐19, I could have seafood and dark chicken soup, which is way more delicious than my daily food. I heard that each patient has a food budget of about US$15 per day. If the food is not enough, they will provide more. Sometimes, the patient could not even finish her meal at all because it was too much. (Lida, quarantined Cambodian garment worker)

The provision of plentiful and nutrient‐rich food during quarantining could be understood in Berlantian terms as a ‘small vacation’ from the grind of hunger and food insecurity. In Berlant's (2007, p. 779) discussion of obesity in the United States, this idea of a ‘small vacation’—an ‘episodic intermission’—is articulated through food as ‘one of the few spaces of controllable, reliable pleasure people have’ and as a result it offers a rare space of pleasure in precarious lives. Garment workers typically spoke of being unable to buy or savour delicious food they enjoyed and focused pragmatically instead on eating ‘just to get full’ so as not to disappoint themselves or their families. To expect food to provide emotional ‘self‐medication’ as Berlant (2007, p. 777) posits, is to inflict an everyday trauma which workers looked to protect themselves from. They described re‐orienting their relationship to food by focusing less on the orosensory pleasures of eating which their lack of food choices largely denied. Here then, but in different ways conceived of originally by Berlant, the body becomes communed in the survival work and production of felt resilience. Garment workers typically cope with economic precarity not through the compensatory pleasures of eating excessively, but rather through the unpleasurable denial of food (Brickell et al., 2022).

## 3 CONCLUSION

What connects these two seemingly divergent phenomena of bodily detriment—impoverished US households reliant on the consumption of unhealthy processed food and Cambodian garment workers dramatically reducing their dietary intake and diversity to the staple of rice—is how food and ‘fates of the body’ are intimately caught up in the market economy. In this sense, as Berlant (2007, p. 774) recognises ‘excess weight and mass hunger are not antithetical states’, but rather symbiotic components of social reproduction under late capitalism. Berlant's work not only establishes then a ‘direct relationship between laboring within capitalism and food consumption, suggesting that how we work, and how capitalism works on us, affects how we eat’, but also asks ‘What is the experience of encountering one's environment in the form of food?’ (Ward, 2013, n.p.). The ‘double crisis’ of unemployment and hunger which garment workers talked to us about during the COVID‐19 pandemic reflected the realities of labouring within a labour‐intensive, low value‐added production regime in which women's bodies and eating habits were shaped by the global capitalism and exploitation of the fashion industry intent on pursuing business‐as‐usual profit‐making in crisis times.

## Data Availability

Access requests to underlying research materials should be addressed to rdm@royalholloway.ac.uk

## Endnote

^1^
See Ward (2013, n.p.) for a critique of Berlant's ‘foundational presumption that consumption drives obesity, that excessive bodies are, in part, produced by excessive consumption, and that fat people consume food people differently than folks that are not fat’. In geography, see also Colls and Evans (2014) for critical discussion.
